# A Study on Refraction Error Compensation Method for Underwater Spinning Laser Scanning Three-Dimensional Imaging

**DOI:** 10.3390/s24020343

**Published:** 2024-01-06

**Authors:** Jinghui Zhang, Yuhang Wang, Tao Zhang, Kai Yang, Jian Zhang, Xinyu Wang

**Affiliations:** Mechanical and Electrical Engineering College, Northeast Forestry University, Harbin 150040, Chinayangkai@nefu.edu.cn (K.Y.); freezeman007@nefu.edu.cn (J.Z.);

**Keywords:** underwater 3D imaging, self-rotating, linear laser scanning, refraction error compensation algorithm, fixed light window and laser spinning (FWLS)

## Abstract

Laser scanning 3D imaging technology, because it can obtain accurate three-dimensional surface data, has been widely used in the search for wrecks and rescue operations, underwater resource development, and other fields. At present, the conventional underwater spinning laser scanning imaging system maintains a relatively fixed light window. However, in low-light situations underwater, the rotation of the scanning device causes some degree of water fluctuation, which warps the light strip data that the system sensor receives about the object’s surface. To solve this problem, this research studies an underwater 3D scanning and imaging system that makes use of a fixed light window and a spinning laser (FWLS). A refraction error compensation algorithm is investigated that is based on the fundamentals of linear laser scanning imaging, and a dynamic refraction mathematical model is established based on the motion of the imaging device. The results of the experiment on error analysis in an optimal underwater environment indicate that the error in reconstructing the radius is decreased by 60% (from 2.5 mm to around 1 mm) when compensating for the measurement data of a standard sphere with a radius of 20 mm. Moreover, the compensated point cloud data exhibit a higher degree of correspondence with the model of the standard spherical point cloud. Furthermore, we examine the impact of physical noise, measurement distance, and partial occlusion of the object on the imaging system inside an authentic underwater setting. This study is a good starting point for looking at the refractive error of an underwater laser scanning imaging system. It also provides to us some ideas for future research on the refractive error of other scanning imaging methods.

## 1. Introduction

In recent years, conventional underwater imaging methods that depend on sonar or stereovision have encountered difficulties as a result of their vulnerability to underwater noise. Consequently, it has become increasingly challenging to attain precise three-dimensional reconstructions of targets submerged in water. As a result, there has been a growing interest in underwater optical three-dimensional reconstruction technology [1]. With the advancement of deep learning, single-image-based 3D reconstruction has made significant progress in underwater imaging. Traditional methods like stereovision have been surpassed by end-to-end training methods that utilize deep learning. These methods can directly take a single image as input and generate a reconstructed 3D model as output. By extracting features more efficiently, the reconstruction effect is improved [2,3]. Nevertheless, this approach does have some drawbacks. Firstly, the available public dataset is limited in size and lacks diversity, leading to a shortage of training data. Additionally, the quality of the reconstructed 3D objects is not particularly impressive in terms of resolution and accuracy. Moreover, obtaining images of underwater low-light environments without an active light source poses a significant challenge. Another prevalent technique for underwater imaging is sonar-based imaging radar, which offers a modest level of resolution and has limitations in capturing intricate details and detecting minute objects. Underwater laser scanning employs laser point cloud scanning technology to precisely capture three-dimensional models of the underwater environment without physical contact. However, because of the refraction that occurs when light passes through different media, it is necessary to apply refraction correction in order to enhance the accuracy of the imaging. This method is well suited for measuring a wide range of underwater scenes and acquiring precise models of targets. Underwater laser scanning imaging serves multiple purposes, including target identification of underwater robots, high-resolution imaging of structures, real-time data assistance for underwater rescue operations, detection of underwater torpedoes, and identification of undersea buildings [4]. In the industrial sector, this technology finds extensive applications in several areas, such as industrial product quality inspection, building analysis [5], water conservancy troubleshooting, and other related sectors [6]. Marine scientific research uses technology to investigate various aspects of the ocean, including the exploration of oil and gas resources beneath the seabed, mapping the topography of the seabed, and the search for submerged archaeological structures of historical significance [7]. Furthermore, due to the advancements and practical implementation of contemporary electronic information technology and underwater imaging technology, the latter is utilized to detect reefs and beacon structures, among other things, and is, thus, employed to some degree in maritime vessel navigation [8]. Additionally, the technology can be utilized to identify the contamination source in contaminated waters [9].

As an active optical measurement technology, three-dimensional line-structured light reconstruction enables the reconstruction of measured targets with high precision [10]. The underlying principle of this methodology entails the application of a laser beam to produce a line, which is then subjected to rotational scanning by a turntable. During this scanning process, the distance and angle of each laser point are meticulously recorded. Consequently, three-dimensional coordinate data pertaining to the target object can be obtained [11]. The linear laser scanning system primarily consists of three major components: a camera (CCD), a linear laser, and a scanning turntable. The calibration of system parameters is a crucial step in achieving accurate three-dimensional reconstruction [12]. This process involves calibrating various parameters, such as the CCD internal and external reference matrices, the light plane equation, and the system rotary axis equation. One of the most important of these parameters is the calibration of the light plane. This is because correct calibration of the light plane equations makes it easier to understand how light moves through water, which allows for accurate adjustments for refraction. Compensating for refraction is an essential process in guaranteeing the precision of the obtained laser scan data. Therefore, precise calibration of the light plane is essential for acquiring point cloud data of superior quality. By conducting camera parameter calibration, it becomes possible to establish the transformation relationship between the pixel coordinate system and the camera coordinate system. By combining this transformational relationship, it is possible to least-squares fit numerous laser strips on the calibration target to derive the plane equation of the light plane in the camera coordinate system. [13]. The calibration of camera parameters and light plane calibration predominantly rely on the Zhang [14] method. This widely used, dependable, and straightforward method finalizes the internal and external parameters of the camera through the extraction of corner points and modeling of the camera using checkboard images captured from multiple angles. However, alternative techniques such as the Dewar method [15], the sawtooth method, and the step measurement method [16] can also yield a specific quantity of calibration points with high precision, facilitating the achievement of camera calibration. Regarding the calibration of the rotary axis, there are several regularly employed methods, namely the cylinder-based approach, the standard ball-based method, and the checkerboard grid calibration method. These methods for calibrating the rotary axis involve observing the measured object to obtain a significant number of highly accurate angle points. The rotational path of these angle points at different heights corresponds to the center of a circle at different heights. By fitting a straight line to the center points of the angle points at different heights, the linear equation of the axis of rotation can be determined.

**Figure 1:**
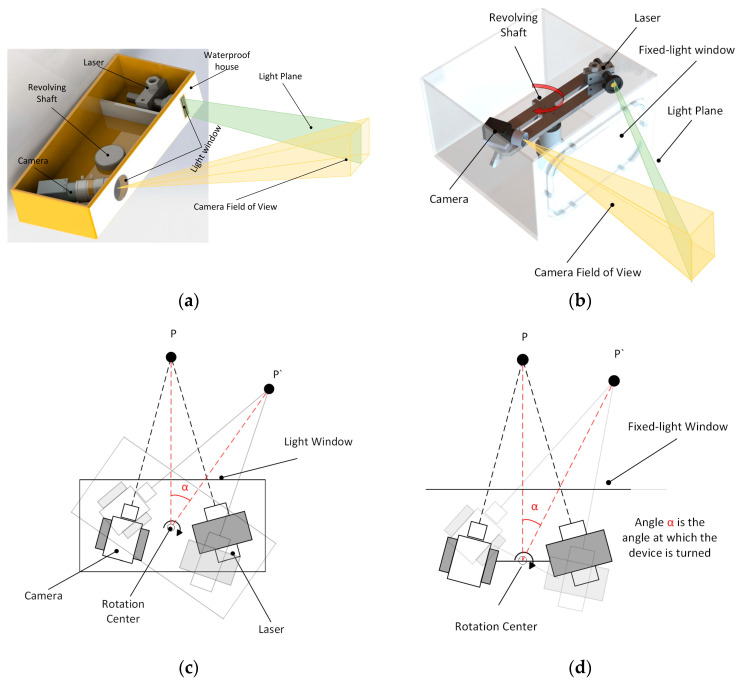
Scanning schematic and equipment schematic of the laser scanning imaging system. (**a**) Common laser scanning imaging system’s scanning schematic; (**b**) FWLS scanning imaging system’s scanning schematic; (**c**) Common laser scanning imaging system’s equipment schematic; (**d**) FWLS scanning imaging system’s equipment schematic.

In the context of addressing the issue of refraction error in a system, as seen in Figure 1a,b, it is common practice in underwater 3D reconstruction to establish a fixed relationship between the light window, laser scanning imaging system, and rotary table. By maintaining a fixed configuration between these components, the system’s refraction error can be analyzed in a more stable manner [17]. As a result, this facilitates the creation of a static compensation algorithm that can be utilized to analyze the refraction process and mitigate the detrimental impacts of refraction on the laser scanning imaging system. The application of this technology facilitates improved observation of the refraction error phenomenon. Nevertheless, the analysis procedure is deficient in specificity and fails to comprehensively consider the mechanical parameters of the imaging system [18]. Furthermore, someone can expand upon the concept of stable refraction by undertaking an exhaustive examination of the two refraction processes through the utilization of three distinct media. In addition, mechanical parameters of the system are taken into account, such as the distance between the window plane and the optical center. This is performed to make the established refraction compensation model [19] more reliable and effective. However, it is worth noting that the calibration method for determining the distance between the optical center and the window plane often heavily relies on the aforementioned compensation model. The complexity of the problem-solving process is noteworthy. In addition to performing an analysis of the refraction process in order to mitigate the inaccuracy, a novel three-dimensional laser sensor was presented by Miguel Castillon et al. [20], wherein the inherent properties of a two-axis mirror were utilized to transform a projected curve into a straight line upon refraction in water. This strategy effectively mitigates the occurrence of refraction errors. In low-light situations underwater, the rotation of a conventional scanning device causes some degree of water fluctuation, which warps the light strip data that the system sensor receives about the object’s surface. To solve the problem, this research studies an underwater 3D scanning and imaging system that makes use of a fixed light window and a spinning laser (FWLS). The refraction compensation algorithm employed in the FWLS scanning imaging system, as investigated in this study, bears resemblance to the refraction compensation method utilized in the galvanometer–fixed light window imaging system [21]. However, it is worth noting that the refraction compensation algorithm in the latter system typically only takes into account the pixel coordinate shift in the return light. As seen in Figure 1b,d schematics, this study presents the development of a model that simulates the dynamic process of a FWLS scanning imaging system based on the imaging principle. The FWLS scanning imaging system’s refraction compensation technique is derived by solving the equations for the light plane and the dynamic pixel coordinate offsets related to dynamic refraction.

In a previous study, the presence of water current and attenuation refraction in water contributed to increased calibration challenges. Wang et al. improved the laser 3D reconstruction method and derived a deep neural network (DNN)-based way to find augmented reality (AR) markers to deal with these problems. An approach to adequately training the model for robustness is described. Upon evaluation, the automatic laser line ID determination reaches 100 percent accuracy, and the detection rate of underwater AR markers reaches a maximum of 91 percent [22]. Chen et al. suggested an underwater stereo-matching algorithm based on a convolutional neural network (CNN) for reconstructing 3D images underwater. This would allow for high framerates even though it would require a lot of computing power. Direct training with unprocessed data significantly reduces the training process’s complexity. To validate the procedure, underwater fish reconstruction experiments were performed employing this approach; the outcomes indicate that the error rate remains below 6% [23]. Nocerino et al. introduced a dense image-matching technique along with a thorough evaluation and analysis of the chosen method. They further validated the accuracy of their algorithm by conducting point cloud analysis on eight distinct objects and scenarios, thereby demonstrating its practicality. Nocerino’s suggested approach for dense image matching produces a point cloud with high density. This point cloud is used to regulate and fine-tune the reconstruction quality of objects based on the dataset’s features. However, the presence of numerous and ambiguous parameters can impact the final outcomes of the reconstruction [24]. Fabio Menna et al. examined the practicality of using photogrammetry to photograph and create 3D models of objects with intricate shapes and optical characteristics. They opted for a semi-automated approach to ensure cost-effectiveness in their experiments, which involved three objects with varying shapes and optical properties. Utilizing a quasi-automatic procedure reduces the expenses of the experiments and produces satisfactory geometries, textures, and accurate color information. However, the method’s high f-numbers, necessary to achieve a sufficient depth of field, lead to excessively long exposure times. Additionally, the experimental equipment is bulky and occupies a significant amount of space, rendering it unsuitable for intricate underwater environments [25]. Thomas Luhmann conducts a comprehensive evaluation and synthesis of the progression of cameras and calibration techniques. He examines several camera-modeling models and places emphasis on current advancements in automatic calibration methods and photogrammetric precision. Self-calibration is a completely automated procedure that is used for both targeted and untargeted objects and scenes. As self-calibration becomes more common, the reliability of calibration is enhanced by increasing the redundancy of observation data. The expense of self-calibration is reduced; however, calibration errors can still inevitably impact the calculation of object points and the independent accuracy of calibrated scenes [26]. Hans-Gerd Maas suggested an automated measurement system that utilizes structured light projection to measure objects with limited surface texture at close distances. He outlined a particular method and tested it on various examples to validate its effectiveness. This method offers all the benefits of photogrammetric systems without the need for operator-provided approximations or initial matches. Nevertheless, this technique is limited to detecting deformation only in the depth coordinate direction. Additionally, the point grating must be physically applied to the surface to accurately compute local strain and shear [27]. Michael Bleier introduced a two-part 3D scanning system designed for shallow water. The technology utilizes satellite navigation and a high-power cross-line laser to extend the detection range. The system is separated into two sections, one above the water and one below. Close-range scanning can produce millimeter-level inaccuracies in experimental settings. The scanning system utilizes a high-power laser that effectively overcomes water absorption and interference from ambient light. The system’s cross-line laser pattern enables an unrestricted scanning motion. However, it is important to note that this system is limited to shallow water detection within the range of 5–10 m. Additionally, the system’s GNSS antenna, located near the lid, provides a low level of localization accuracy, which can result in errors [28]. In the pursuit of investigating an algorithm for compensating underwater refraction errors, one approach is to make modifications to the existing method in order to minimize errors [29]. Alternatively, a novel correction algorithm can be developed with the specific objective of addressing refraction-related distortions [30]. Hao [31] and Xue developed a refraction error correction algorithm based on their system’s refraction model. This technique effectively enhances the three-dimensional image accuracy of the system to approximately 0.6 mm. In their study, Ou et al. [32] employed a combination of binocular cameras and laser fusion technology to develop a model of the system. They conducted an analysis of the refraction error, performed system calibration, and ultimately achieved high-precision imaging in low-light underwater conditions.

However, the imaging modes of the systems examined by Hao, Xue, Ou, et al. exhibit fluctuating effects on the water body during scanning. This causes distortion in the light bar information on the surface of the measured object, resulting in undesired reconstruction errors. Therefore, this paper focuses on studying the FWLS scanning and imaging system, as depicted in Figure 1d. In conjunction with Hao, Xue, et al.’s refraction error compensation algorithm, this study develops a mathematical model to represent the dynamic refraction process of the FWLS scanning imaging system. As a result, this paper offers a dynamic refraction error compensation algorithm to address the fluctuation error issue that underwater rotational scanning causes in water bodies. By doing so, this paper aims to circumvent the fluctuation error problem that arises during the system’s rotational scanning in low-light conditions underwater.

## 2. Description of the Underwater Imaging Device

**Figure 2:**
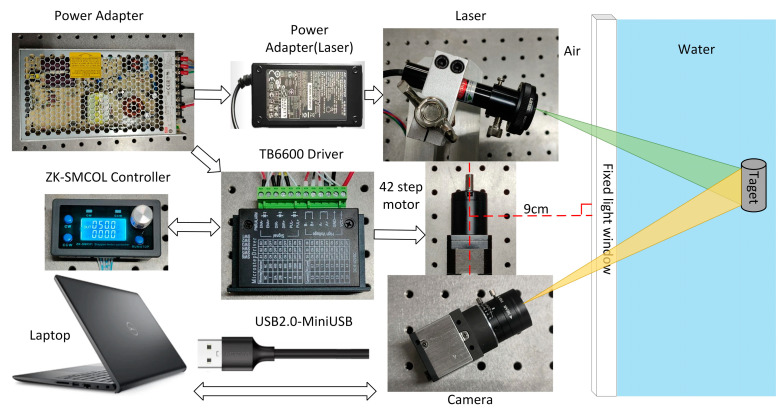
Hardware components of homemade underwater imaging device.

As depicted in Figure 2, the hardware component of the underwater imaging equipment comprises a CCD camera, a linear laser, a rotating table, a controller, and a driver. The camera utilized in this study is Thorlabs’ DCU224C industrial camera (Optical equipment, Newton, NJ, USA), which offers a resolution of 1280 × 1024 pixels. It operates within a spectral range of 350 nm to 600 nm. The camera is equipped with an 8 mm focal length lens, specifically the MVCAM-LC0820-5M model (Lankeguangdian Co. Ltd., Hangzhou, China), providing a field-of-view angle of 46.8° horizontally, 36° vertically, and 56° diagonally. For the linear laser, a 520 nm linear laser with a power output of 200 mW was selected. The rotary table employed in this setup consists of a 42-step motor, along with its corresponding controller and driver.

**Figure 3:**
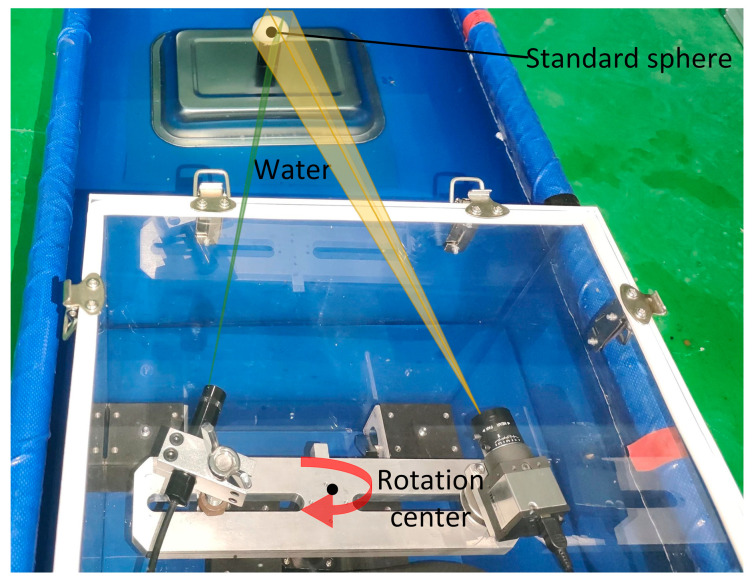
Physical drawing of underwater imaging device.

As seen in Figure 3, the camera and laser are affixed to opposite ends of the rotary table beam, with their relative locations remaining constant. The motor rotation axis is positioned at a vertical distance of 9 cm from the light window. By manipulating the motor rotation speed and direction at the controller end, the beam may be rotated. This allows for horizontal scanning of the system at a fixed point within a 360° range. The complete device is housed within a waterproof cover constructed from ultra-clear transparent glass, which has a transmittance rate of 90%, with dimensions of 30 cm × 30 cm × 40 cm and a wall thickness of 5 mm. The refractive index of water is 1.3333.

The system workflow is as follows: initially, the system camera, light plane, and rotation axis are calibrated in the atmosphere. Then, the underwater refraction error compensation algorithm is integrated into the 3D point cloud-stitching program. When the system scans the measured object, the controller rotates the rotary table at the predetermined rotational speed. Simultaneously, the camera captures images of the light strip during the rotation scanning process. After the scanning is finished, the captured images are processed on a PC to obtain the image coordinates of the structure’s light center. Once the scanning is finished, the obtained image is analyzed on the computer to determine the precise coordinates of the center of the structural light. The coordinates are transformed to derive the spatial coordinates of the object’s surface being examined. After making a dynamic refraction adjustment, the 3D point cloud model of the object is made by combining the 3D point cloud-stitching method with the system’s rotational speed.

### 2.1. The Calibration of Light Planes and Rotation Axes

#### 2.1.1. Light Plane Fitting Utilizing the Least-Squares Method

**Figure 4:**
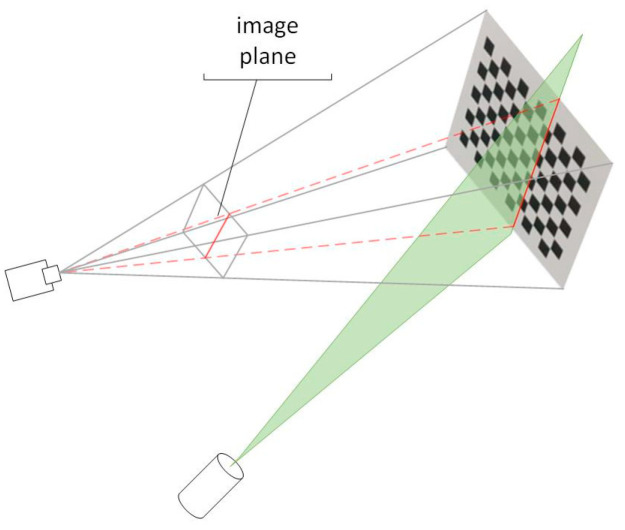
Schematic diagram of light plane calibration.

The CCD internal and external reference matrices, as well as the transformation matrix between the camera coordinate system and the tessellated coordinate system [33], can be readily derived using the Zhang calibration method. As seen in Figure 4, if ax+by+cz=d is the light plane equation, then finding the coefficients of this plane equation only requires four locations. Using the checkerboard calibration method, first create two sets of checkerboard calibration images, one with and one without light strips. Next, use the light strip extraction method in Section 3 to extract the actual coordinates of the light strips on the target. Finally, use the conversion matrix to convert the coordinates to the camera coordinate system. From the two sets of images, two linear equations can be extracted, and the light plane equation can be found using least-squares fitting [34,35].

Error equation from plane equation:

(1) $$s=\sum_{i=1}^{N_{1}}(ax_{i}+by_{i}+cz_{i}{)}^{2}$$

By streamlining the error equation’s partial derivation, we can obtain:

(2) $$\begin{array}{c} \sum_{i=1}^{N1}\left(ax_{i}^{2}+bx_{i}y_{i}+cx_{i}z_{i}+x_{i}\right)=0\\ \sum_{i=1}^{N1}\left(ax_{i}y_{i}+by_{i}^{2}+cy_{i}z_{i}+y_{i}\right)=0\\ \sum_{i=1}^{N1}\left(az_{i}x_{i}+by_{i}z_{i}+cz_{i}^{2}+z_{i}\right)=0 \end{array}$$

Since the system of equations is linear, Clem’s approach can be used to solve it and obtain the final coefficients of the plane equation as:

(3) $$a=\frac{D_{1}}{D},b=\frac{D_{2}}{D},c=\frac{D_{3}}{D}$$

In conclusion, the light plane equation that results from the 40 cm measurement distance’s experimental calibration is:

(4) $$\left[x\;y\;z\;1\right]\left[\begin{array}{c} \begin{array}{c} -0.7467\\ 0.01058\\ 0.6649 \end{array}\\ 12.2539 \end{array}\right]=0$$

#### 2.1.2. The Calibration of Rotary Axis

**Figure 5:**
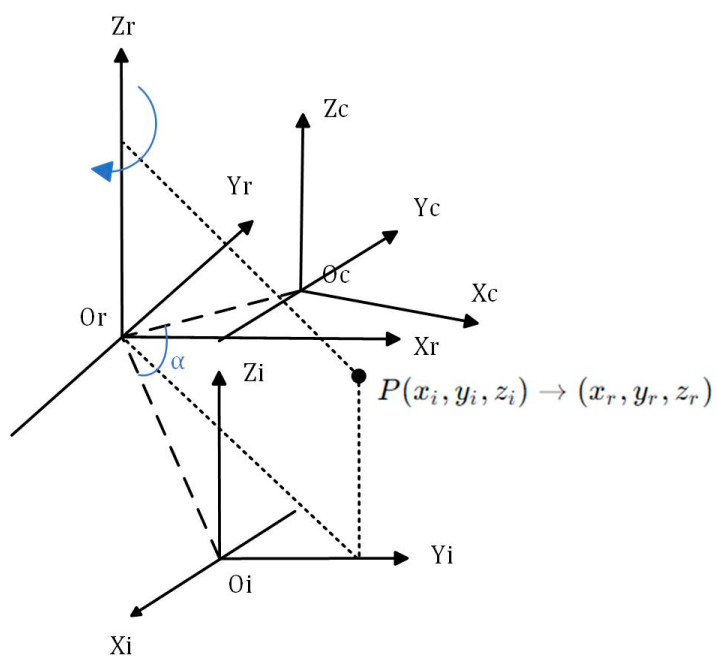
Theoretical model of the imaging device.

In the context of rotary scanning measurement, if the rotation angle is known (which is determined by the motor controller), it is possible to obtain the point cloud data of the entire object surface by performing a coordinate solution using the camera external reference matrix and the light plane equation, provided that the linear equation of the rotation axis is obtained. As depicted in the theoretical model of the imaging device illustrated in Figure 5, it is observed that the *Zr* axis aligns with the rotational axis of the system. The coordinate system for the rotation axis is denoted as $X_{r}O_{r}Y_{r}$.The camera coordinate system at the initial scanning location of the device is denoted as $X_{c}O_{c}Y_{c}$, while the CCD optical center is represented as *Oc*. After rotating clockwise by an angle of α around the rotation axis *Zr*, the camera coordinate system is denoted as $X_{i}O_{i}Y_{i}$.

**Figure 6:**
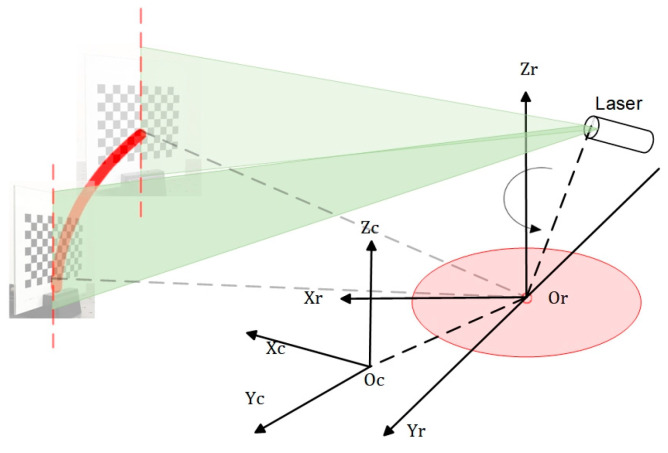
Schematic diagram of rotary axis calibration.

As depicted in Figure 6, the schematic figure illustrates the process of rotary axis calibration. It is imperative that during the rotation and scanning of the device, each point on the target’s trajectory must be on a circle centered on the axis of rotation. As a result, the checkerboard grid’s corner points that rotate at a specific angle are first calibrated using the Zhang method. The corner points’ coordinates are then converted to the camera coordinate system, and they are subsequently fitted to a circle. Subsequently, the property that the angle points of various heights on the chessboard target are placed at different places on the rotation axis is used to generate a series of coordinates of the center of a circle. Finally, the circle’s centers’ coordinates are fitted to a straight line, allowing the equation of the straight line for the rotary axis in the camera coordinate system to be obtained as $a_{r}x+b_{r}y+c_{r}z=d$ [36,37].

The conversion of pixel coordinates to the rotary axis coordinate system is performed in the following manner:

(5) $$\left[\begin{array}{c} x_{r}\\ y_{r}\\ z_{r} \end{array}\right]=RA^{-1}\left[\begin{array}{c} u\\ v\\ 1 \end{array}\right]+T$$

where *A* is the internal reference matrix of the camera and *R* and *T* are the rotation and translation matrices from the pixel coordinate system to the rotational coordinate system; thus, obtaining the matrix *A* is as simple as:

(6) $$A=\left[\begin{array}{ccc} 1806.73 & 0 & 0\\ 0 & 1806.51 & 0\\ 634.62 & 513.50 & 1 \end{array}\right]$$

The values 1806.73 and 1806.51 in matrix *A* represent the focal length, which signifies the ratio between the pixels and the actual length in the horizontal and vertical directions of the image. The values 0 and 0 in the third column of the matrix represent the principal point of the image, indicating the pixel coordinates of the image’s center. The values 634.62 and 513.50 in matrix *A* correspond to the first- and second-order coefficients of the camera’s radial distortion, respectively. Additionally, a value of 1 indicates the distortion of the tangential. In conclusion, the equation for the plane of the rotary axis derived from the 40 cm measurement distance’s experimental calibration is:

(7) $$\left[x\;y\;z\;1\right]\left[\begin{array}{c} \begin{array}{c} 0.4999\\ 37.1895 \end{array}\\ -1\\ 108.6590 \end{array}\right]=0$$

The CCD optical center exhibits circular motion with the rotating shaft as its center. According to the aforementioned calibration, the vertical distance between the CCD optical center coordinate and the rotating shaft is determined to be *R* = 109.7855 mm. The measured distance between the center of the rotating axis and the center of the camera is approximately 110 mm, which closely aligns with the calibration findings of the rotary axis in determining the value of *r*. The credibility of the calibration data pertaining to the rotational axis is evident.

## 3. Laser Strip Center Extraction and Point Cloud Construction

Rapid and precise extraction of the laser strip centerline of the measured object surface is required to achieve high precision of the laser scanning reconstruction system, as the laser strip reflects target surface shape information.

**Figure 7:**
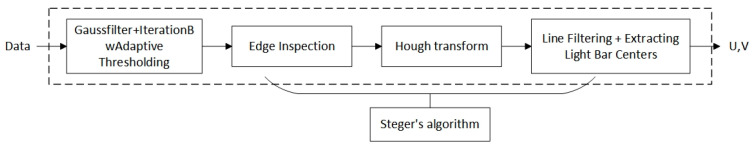
Flowchart of the light bar center extraction algorithm.

**Figure 8:**
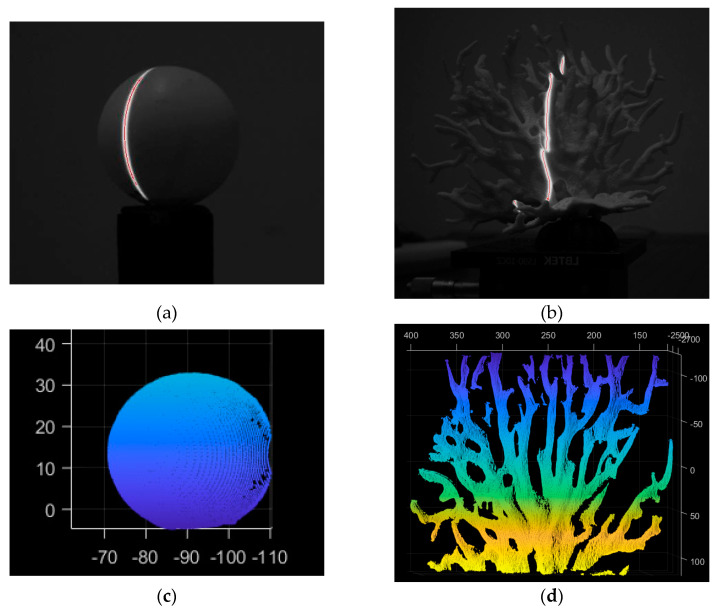
Diagram of the light bar center extraction effect. (**a**) Extraction effect of standard sphere stripe center; (**b**) Extraction effect of coral stripe center; (**c**) 3D point cloud model of a standard sphere; (**d**) 3D point cloud model of coral.

The flowchart of the light bar center extraction algorithm used in this paper is shown in Figure 7. In order to obtain a smoother denoised image, we first apply Gaussian filter denoising, which involves summing up all of the image’s pixel values and dividing each value by itself as well as by the values of the other pixels in the area around the weighted average of the value. Next, the IterationBw algorithm updates the adaptive threshold for light strip extraction, and the Steger algorithm extracts the light strip’s center point [38,39,40]. This method makes it possible to rapidly and accurately acquire the light bar center coordinates of the scanning data. Figure 8a,b illustrates the extraction impact during the actual measurement.

(8) $$\left\{\begin{array}{c} Z_{C}\left[\begin{array}{c} u\\ v\\ 1 \end{array}\right]=\left[\begin{array}{ccc} \frac{1}{dx} & 0 & u_{0}\\ 0 & \frac{1}{dy} & v_{0}\\ 0 & 0 & 1 \end{array}\right]\left[\begin{array}{cccc} f & 0 & 0 & 0\\ 0 & f & 0 & 0\\ 0 & 0 & 1 & 0 \end{array}\right]\left[\begin{array}{cc} R & T\\ \vec{0} & 1 \end{array}\right]\left[\begin{array}{c} X_{W}\\ Y_{W}\\ Z_{W}\\ 1 \end{array}\right]=\left[\begin{array}{cccc} f_{x} & 0 & \nu_{0} & 0\\ 0 & f_{y} & \nu_{0} & 0\\ 0 & 0 & 1 & 0 \end{array}\right]\left[\begin{array}{cc} R & T\\ 0 & 1\\ 0 & 1 \end{array}\right]\left[\begin{array}{c} X_{W}\\ Y_{W}\\ Z_{W}\\ 1 \end{array}\right]\\ ax+by+cz=d \end{array}\right.$$

In Equation (8), *Zc* signifies the Z coordinate of the observed point on the camera’s optical axis, i.e., the object’s depth or distance in the camera’s coordinate system. *dx* and *dy* are the unit pixel’s physical dimensions in the X and Y axes; *fx* and *fy* are the camera’s focal point coordinates; and *R* and *T* are the translation and rotation matrices from the camera coordinate system to the world coordinate system. After obtaining the two-dimensional pixel coordinates of the center of the light plane, the light plane and coordinate system transformation connection found in Section 2.1.1 and 2.1.2 can be combined to acquire the point cloud data of the observed target, as indicated in Formula (8). The system’s motion parameters (motor speed) are then inserted, and the 3D point cloud data are stitched to produce the 3D point cloud model of the observed target, as shown in Figure 8c,d.

## 4. Refractive Error Compensation in Underwater Light Windows

**Figure 9:**
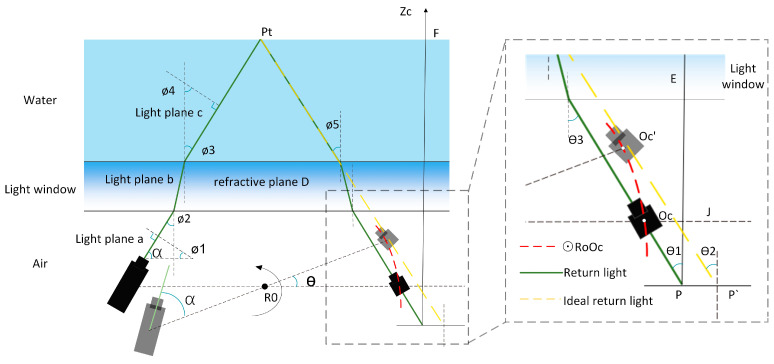
Refractive error modeling of FWSL scanning imaging devices.

In the context of underwater measurements, the FWLS scanning imaging device is enclosed within a waterproof cover to ensure its functionality. Consequently, the device and the object being measured are situated in distinct media. When the instrument is operational, the laser is emitted towards the object being measured by passing through the light window and water. The light that is reflected back from the object likewise passes through the light window and water and is subsequently detected by the CCD. Hence, it becomes apparent that the light plane and the CCD image coordinate experience an excursion due to refraction [41,42,43]. To address this issue, mathematical models are established based on the measurement process, allowing for the determination of equations describing the dynamic light plane refraction and the offset of pixel coordinates on the plane. This mathematical model is illustrated in Figure 9.

As indicated in Figure 9, where Ro is the rotating axis’s center, angle$\;\emptyset_{1}$ represents the inclination between the normal vector of the light plane a and the horizontal direction, while angle$\;\emptyset_{4}$ denotes the inclination between the light plane c and the vertical direction. $\emptyset_{2}$ denotes the angle at which the laser light enters the light window, while $\emptyset_{3}$ represents the angle at which the laser light exits the light window and enters the water. Similarly, $\emptyset_{5}$ signifies the angle at which the return light enters the light window from the water, and $\theta_{3}$ denotes the angle at which the return light exits the light window and enters the air. P’ is the coordinate point on the image plane of the theoretical return light of the measured target point Pt, and position P is the coordinate point on the image plane of the actual return light of the measured target point Pt. The angle formed by the laser and the rotating table’s beam is α. When the device is in its original position, the CCD is at position *Oc*, and it rotates counterclockwise for t seconds to reach position *Oc’*, with a rotation angle of θ. The simple geometric connection can be used to calculate the relationship between $\emptyset_{2}$ and the angle of rotation at moment t:

(9) $$\emptyset_{2}=\alpha +\theta -90{}^{\circ}$$

The variable θ can be mathematically represented as (N−1)Tω, where *N* is the number of frames associated with the image, *T* represents the camera frame rate, and ω signifies the minimum rotation unit speed of the device.

### 4.1. Resolving the Light Plane’s Dynamic Refraction Equation

Let us consider a refracting plane with a normal vector D(0,0,1). The incident light plane, with a normal vector *(a*, *b*, *c)* denoted as (a), is refracted through the glass and forms a new light plane, denoted as (b), with a normal vector ($a^{\prime }$, $b^{\prime }$, $c^{\prime }$). Subsequently, the light plane b enters the water through the glass and is refracted again, resulting in a new light plane denoted as (c), with a normal vector ($a^{\prime \prime }$, $b^{\prime \prime }$, $b^{\prime \prime }$). The refractive index ratio between air and water is denoted as $n_{w}$. By Snell’s law:

(10) $$n_{w}=\frac{n_{a}}{n_{c}}=\frac{\sin{ø}_{2}}{\sin{ø}_{3}}=\frac{\cos{ø}_{1}}{\cos{ø}_{4}}$$

(11) $$\frac{D}{\sqrt{a^{2}+b^{2}+c^{2}}}=n_{w}\frac{D}{\sqrt{a^{\prime \prime 2}+b^{\prime \prime 2}+c^{\prime \prime 2}}}$$

Normalize the light plane c’s normal vector:

(12) $$\sqrt{a^{\prime \prime 2}+b^{\prime \prime 2}+c^{\prime \prime 2}}=1$$

Putting this into Equation (11) results in:

(13) $$D^{\prime }=n_{w}\frac{D}{\sqrt{a^{\prime \prime 2}+b^{\prime \prime 2}+c^{\prime \prime 2}}}$$

We know that since the normal of the light plane (*a*), the refraction plane (*D*), and the refracted light plane (*c*) are coplanar:

(14) $${\left[\begin{array}{c} a\\ b\\ c \end{array}\right]}^{T}=x{\left[\begin{array}{c} a^{\prime \prime }\\ b^{\prime \prime }\\ c^{\prime \prime } \end{array}\right]}^{T}+y{\left[\begin{array}{c} 0\\ 0\\ 1 \end{array}\right]}^{T}$$

Equations (13)and (14) provides us with:

(15) $$\left\{\begin{array}{c} a^{\prime \prime }=\sqrt{\frac{a^{2}{n_{w}}^{2}(a^{2}+b^{2}+c^{2})-a^{2}c^{2}}{{n_{w}}^{2}(a^{2}+b^{2}+c^{2})(a^{2}+b^{2})}}\\ b^{\prime \prime }=\frac{a^{\prime \prime }b}{a} \end{array}\right.$$

If we substitute the intersection point of the light plane (*c*) with the light window $\left(0,(H+f)\cot\emptyset_{2},H+f\right)$ into the equation for the light plane (*c*),where *H* is the distance from the CCD optical center to the light window, we obtain:

(16) $$\left\{\begin{array}{c} b^{\prime \prime }(H+f)\cot\emptyset_{2}+C^{\prime \prime }(H+f)+d^{\prime \prime }=0\\ b(H+f)\cot\emptyset_{2}+C(H+f)+d=0 \end{array}\right.$$

In conclusion, only the distance between the CCD optical center and the glass is unknown. The CCD optical center rotates in a circular motion with Ro serving as the center and R as the radius from the *Oc* position to the *Oc’* position, as seen in Figure 9’s right panel. The equation for the circle with center *Ro* and radius *r*, denoted as ⊙*RoOc*, can be derived as ${x_{r}}^{2}+{z_{r}}^{2}=r^{2}$. When the device is mounted, the distance from the spinning shaft’s center to the light window–water side is 90 mm, and the equation of the line of refraction *D* is *z* = 90. It is easy to obtain $H=\sqrt{r^{2}-{x_{r}}^{2}}-90$. Next, by utilizing Equation (5), the H expression may be converted to align with the camera coordinate system. Consequently, the plane equation of the refracted light plane (c) can be derived by employing the coupling Equations (15) and (16).

### 4.2. Solution for the Pixel Coordinate Offset Coefficient

The schematic picture in Figure 9 illustrates the mapping of the underwater target point Pt onto the image plane, resulting in the imaging point *P (u, v).* If the return light is not subject to refraction by the water body and the light window, it is observed directly at the location of $P^{\prime }$. By determining the offset coefficient η between the two points, it is possible to achieve refraction correction for each measured point [44,45,46,47,48]. The offset η, which represents the difference between the point $P^{\prime }$ on the image plane corresponding to the theoretical return light and the point *P* on the image plane corresponding to the actual return light, can be mathematically described as the ratio of the tangent of $\theta_{1}$ to $\theta_{2}$, as seen in Figure 9.

(17) $$\eta =\frac{\tan\theta_{1}}{\tan\theta_{2}}=\frac{tan\theta_{3}}{\tan\theta_{2}}=\frac{tan\theta_{3}}{O_{c}J/PJ}$$

also known as:

(18) $$\begin{array}{c} IJ=\sqrt{u^{2}+v^{2}}\\ PJ=f\\ O_{c}J=\sqrt{u^{2}+v^{2}}\\ PJ=f \end{array}$$

where *f* is the camera’s focal length. By Snell’s law: $\tan\theta_{3}=\tan\emptyset_{5}=\tan\emptyset_{3}=\tan\emptyset_{2}$.

Then bring Equation (9) into (17) to obtain:

(19) $$\eta =\frac{\tan\left[arcsin(\frac{\sin\left(\alpha +\theta -90\right)}{n_{w}})\right]}{\sqrt{u^{2}+v^{2}}/f}$$

that is:

(20) $$\eta =\frac{\tan\left[arcsin(\frac{\sin\left(\alpha +\left(N-1\right)T\omega -90\right)}{n_{w}})\right]}{\sqrt{u^{2}+v^{2}}/f}$$

## 5. Error Analysis Experiments

To evaluate the precision of the system, we conducted multiple scans and reconstructions of a standard ball with a radius of 20 mm. This was performed within a range of 30 cm to 80 cm, with *D* representing the working distance. Additionally, we selected six positions within the imaging field of view at different distances. The objective was to calculate the measurement radius of the standard ball before and after correcting for refraction errors. The radius of the standard sphere measurement underwater, without the use of the refraction compensation method, is represented by the symbol *R*. Conversely, the radius of the measurement after accounting for the correction of refraction errors is marked by *Rw*. The radius of the standard ball can be determined through a computation, and the measured radius of the standard ball is presented in the table provided.

**Figure 10:**
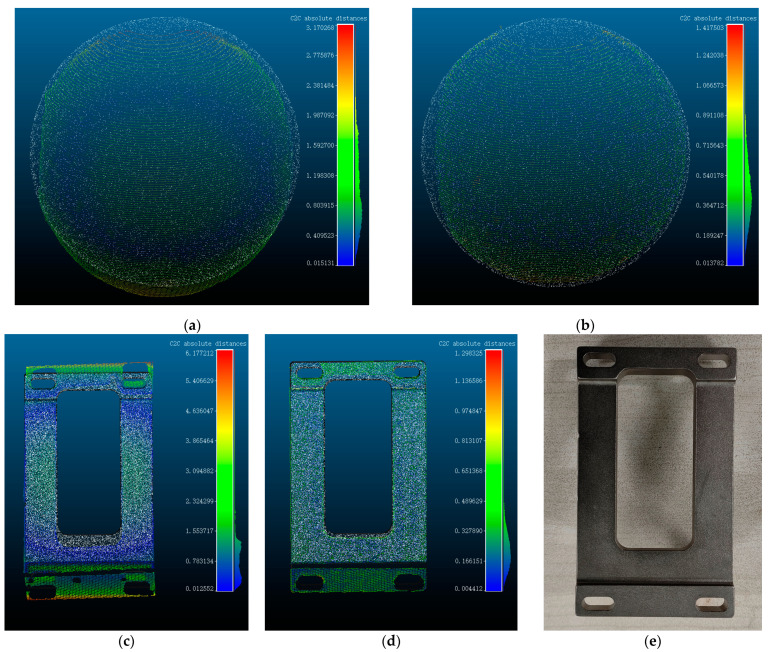
Point cloud error plotted before and after refraction error compensation. (**a**) The distance between the point cloud before refraction error compensation and the standard sphere point cloud. (**b**) The distance between the refraction error compensation point cloud and the standard spherical point cloud. (**c**) Distance between point clouds before correction of standard workpieces. (**d**) Distance between point cloud correction of standard workpieces. (**e**) Measured standard workpieces (approximate dimensions of workpiece: 101 mm × 60 mm × 7 mm).

As seen in Table 1, in the absence of the refraction error compensation method, the measurement error of the standard ball remains within a range of 2.5 mm. The largest measurement error observed is 2.36 mm, while the minimum measurement error is −0.67 mm. Upon the implementation of the refraction error compensation algorithm, the reconstructed standard ball radius exhibits a minimum error of 0.18 mm and a maximum error of 0.9 mm. It is apparent from this observation that the addition of the refraction error correction method improved the system’s reconstruction accuracy to some extent. Subsequently, the point cloud data obtained before and following the application of refraction adjustment were chosen for comparison with the point cloud data of the standard ball, which has a radius of 20 mm. The distances between the point clouds are illustrated in Figure 10a,b.

The varying hues of the right-side bars in Figure 10a–d correspond to distinct values that indicate the disparity between the measured point cloud model and the point cloud of the standard workpiece or standard sphere. The point cloud of the standard workpiece or standard sphere is depicted in white, while the colored point cloud represents the data obtained from the actual measurement. The color gradient from blue to red signifies the range of distances, with blue indicating proximity and red indicating greater distance. The measured point cloud with the addition of the refraction compensation method clearly matches the actual standard sphere model better, as shown in Figure 10a,b, and the overall curvature and other details are enhanced. To further validate the efficacy of the compensation method, we conducted scanning reconstruction of the workpiece depicted in Figure 10e, and a satisfactory compensation result is observed as shown in Figure 10c,d.

**Figure 11:**
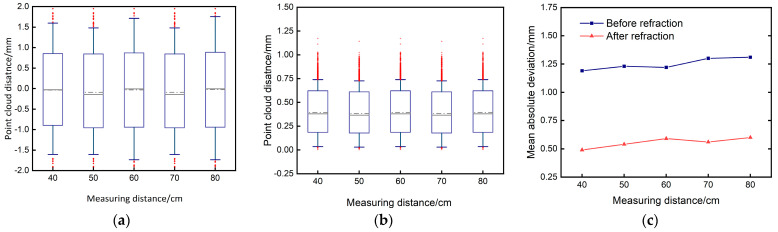
Point cloud error distribution diagram. (**a**) Before compensation, the distribution of distances between the point cloud and the standard sphere. (**b**) Distance distribution map between the compensated point cloud and the standard ball. (**c**) Mean absolute deviation of point cloud distance before and after compensation.

Five sets of point clouds were chosen, both before and after compensation. These point clouds were registered with the standard sphere point cloud, and the distances between the five groups of point clouds and the standard sphere point cloud were obtained. The box plots regarding distance distribution are presented in Figure 11a–c. The box plot diagrams reveal that the disparity between the measured point cloud and the standard sphere is primarily concentrated within the range of (−1, +1) prior to compensation. However, after compensation, the disparity is predominantly distributed within the range of (0.25, 0.75). The mean absolute error of the distance between the acquired point clouds is computed, as depicted in Figure 11c. The mean absolute deviation (MAD) prior to compensation measures approximately 1.25 mm. However, after applying the refraction compensation method, the MAD is effectively lowered to around 0.5 mm. This observation substantiates the authenticity and efficacy of the compensation algorithm. The analysis of dynamic refraction compensation serves as a foundation for future research on approaches for compensating dynamic refraction.

The first two columns of Table 2 display the two frequently employed underwater laser scanners, while the last two columns showcase the scanning modes extensively utilized in contemporary academic research. The SXLS-100 product (Lankeguangdian Co. Ltd., Hangzhou, China) [49] is identical to the scanning imaging device studied by Xue et al., and the ULS-100/200 product (Voyis Imaging Inc. Waterloo, ON, Canada) [50] is identical to the scanning imaging device studied by Xie et al. Xie’s mode combines galvanometer scanning with a fixed camera and fixed light window, whereas Xue’s mode is illustrated in Figure 1a. Compared to industrial products, the resolution and accuracy of the homemade imaging rotary in this paper are similar within a certain measurement distance. However, there is still a gap between the working distance and scanning line refresh rate. This is because the scanning mode of FWLS is constrained by the mechanical characteristics of the rotation mechanism, preventing it from achieving a very fast scanning speed. Additionally, when the imaging distance is large, the angle between the optical axis of the camera and the laser line becomes too small, resulting in fewer captured image pixels and lower scanning resolution. However, increasing the distance between the camera and the laser can extend the working distance of the imaging system. The FWLS scan line refresh rate is comparatively lower in magnitude than the galvanometer scanning approach investigated by Xie et al. However, the FWLS scanning imaging transposition manages to attain a satisfactory working distance and imaging accuracy, all while preserving a low cost of ownership. In contrast to the conventional scanning mode depicted in Figure 1a, investigated by Xue et al., the imaging device examined in this study guarantees an equivalent scan line refresh rate while achieving superior accuracy and working distance. Furthermore, the scanning imaging mode of FWLS effectively prevents the distortion of the obtained light strip data resulting from the fluctuation of water generated by the rotational scanning of the system. Simply put, when compared to current industrial goods and scanning imaging devices offered in academic research, the FWLS system achieves a commendable level of resolution and accuracy. However, its working distance and scan line refresh rate are rather average. The imaging precision of FWLS can be enhanced by selecting a camera with a higher pixel resolution, a linear laser with a narrower light bar, and a mechanical rotation mechanism with reduced mechanical errors. Similarly, the imaging range of FWLS can be expanded by increasing the distance between the camera and the laser.

The mechanical error of the rotary table and other structures must be minimized if we are to achieve a more rapid 3D reconstruction of targets. Additionally, the volume and weight of the rotating hardware, including cameras and lasers, should not be excessively large. Mechanical scanning has certain limitations that restrict the attainment of extremely high scanning speeds to the more expensive galvanometer scanning, memes, and other imaging modes. Nonetheless, the FWLS scanning imaging scheme investigated in this article is applicable to general underwater low-light scenarios, is inexpensive, and can achieve a respectable scanning speed while preserving a degree of imaging precision. Additionally, the turbidity, fluctuations, and presence of aquatic organisms in the water will have a significant impact on the imaging accuracy in challenging subaqueous settings. Therefore, to accurately replicate the underwater environment, we have examined the impact of physical noise, imaging distance, and partial obscuration of the measured target on the system.

### 5.1. Underwater Physical Noise’s Effects on the System

To replicate the effects of actual underwater sediment, suspended solids, and other physical disturbances on the system, we introduced a specific quantity of sediment and milk into 132 L of fresh water. Subsequently, we conducted experiments to assess the influence of varying levels of turbidity in the underwater environment on the imaging system.

**Figure 12:**
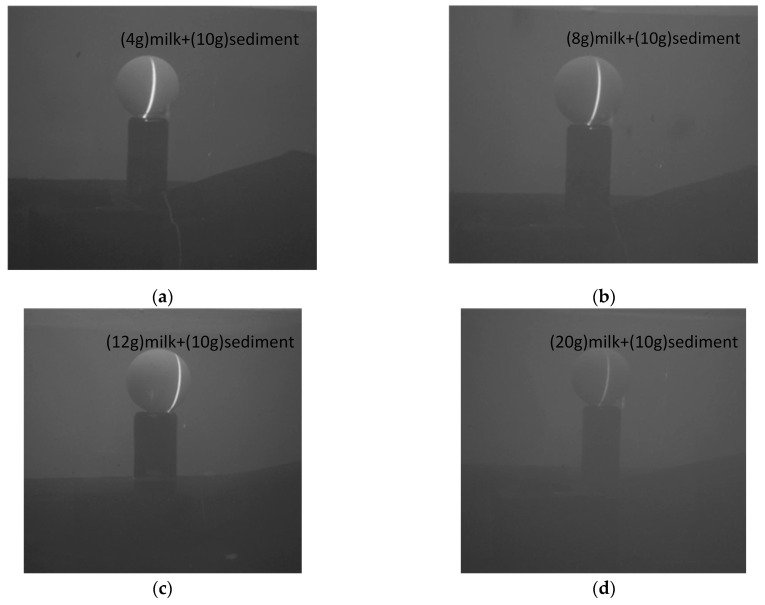
Diverse turbidity of underwater environments. (**a**) Image showing water’s turbidity after adding 4 g of milk and 10 g of sediment. (**b**) Image showing water’s turbidity after adding 8 g of milk and 10 g of sediment. (**c**) Image showing water’s turbidity after adding 4 g of milk and 12 g of sediment. (**d**) Image showing water’s turbidity after adding 4 g of milk and 20 g of sediment.

**Figure 13:**
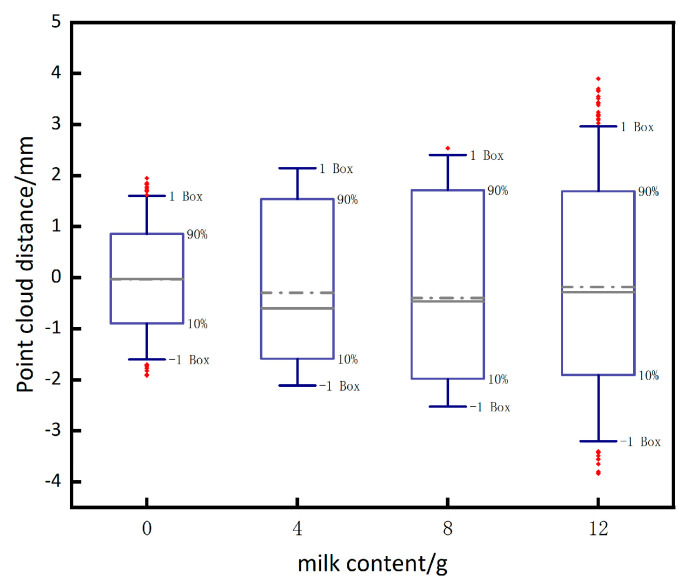
The impact of varying underwater physical noise concentrations on system.

As shown in Figure 12, different amounts of milk and sediment were added to the experimental pool to create underwater environments with varying turbidity, and measurements were taken on the standard sphere in each of these four environments to obtain the error corresponding to the (a), (b), (c) plots as shown in Figure 13. When 20 g of milk is added to the underwater, the underwater turbidity is too high, resulting in the inability to image the underwater. To summarize, excessive turbidity in underwater conditions has two main effects on imaging. Firstly, it causes the scattering of light in both forward and backward directions, which hampers the quality of the images. Secondly, suspended particles in the water, like silt, partially absorb the returning light, leading to an inability to capture clear images.

### 5.2. Impact of Measuring Distance on System

**Figure 14:**
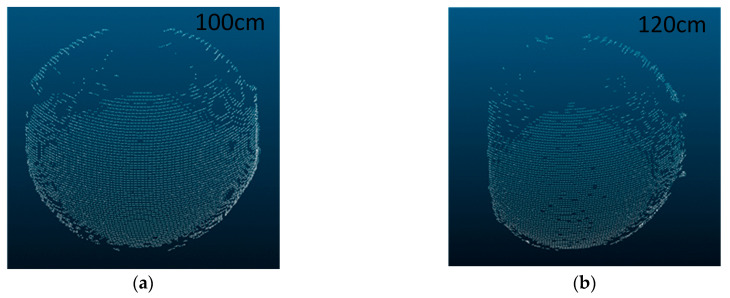
Effect of larger measurement distances on imaging. (**a**) Measuring imaging at a distance of 100 cm. (**b**) Measuring imaging at a distance of 120 cm.

As shown in Figure 10b and Figure 14, when the distance being measured is large, the water body absorbs light, causing the CCD to receive inadequate return light. As a result, the scanned photographs taken have lower contrast, leading to partial missing data in the measurements. When the system approaches the maximum measurement distance, the small angle between the system’s optical axis and the light plane causes fewer pixels to be scanned in the image. This leads to lower resolution in the scanning results. At this point, the system’s error becomes the main factor and the impact of refraction compensation is less significant. Finally, based on the data acquired from the 120 cm and 100 cm measurement distances in Figure 14, it is obvious that a measuring distance that is too far away primarily affects the reception of the backhaul light, resulting in missing data. Therefore, in order to ensure accurate measurement data, it is crucial to keep the measurement distance within the system’s working range.

### 5.3. Effect of Partial Occlusion of the Measured Target on the System

**Figure 15:**
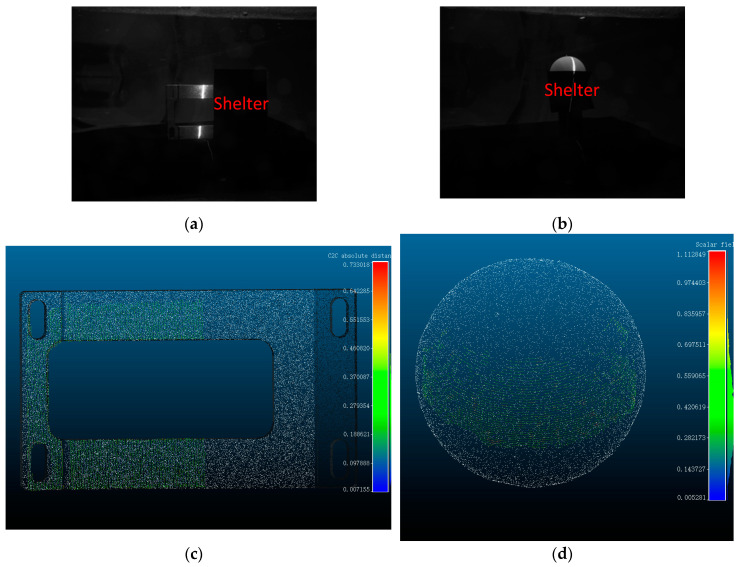
Simulation and error analysis of a measured target partially covered by an underwater barrier. (**a**) Underwater partially obscured standard workpieces. (**b**) Underwater partially obscured standard ball. (**c**) Underwater partly shaded standard workpiece error analysis. (**d**) Underwater partly shaded standard ball error analysis.

Figure 15 demonstrates our use of a shade cloth to imitate an underwater obstacle obstructing the measured target. When the underwater target is obstructed by another obstacle, the occluded portion cannot be measured. However, the unobstructed portion follows the refraction compensation algorithm as usual. Scenarios involving the occluded light plane and the occluded light path of the camera’s return path are analogous to this case.

## 6. Conclusions

This study presents a comprehensive description of an advanced underwater fixed light window and laser spinning scanning 3D imaging system. The system is designed to achieve enhanced accuracy in acquiring 3D point cloud data of underwater targets. It accomplishes this through the use of a green linear laser, a CCD camera, a rotary table, and a drive unit. This study employs a meticulous calibration process for the light plane and rotary axis to guarantee the precision and correctness of the collected data. The extraction of point cloud data involves the utilization of the Steger method, which is complemented by Gaussian filtering and iterative binarization techniques. This combination is employed to achieve a superior level of accuracy and precision in the light strip center data extraction process. Furthermore, this research presented a novel technique for compensating refraction errors caused by the dynamic refraction process induced by the laser passing through different media in the fixed light window and laser spinning scanning 3D imaging system. Experimental evidence substantiated the efficacy of this algorithm in enhancing the accuracy of the collected data. The experiments involved conducting an error analysis utilizing a standard sphere. The findings indicated that the reconstruction error ranged from 2.5 mm when the refraction error compensation was not applied. However, after implementing the compensation algorithm, the error reduced to less than 1 mm, resulting in the attainment of highly precise point cloud data. Furthermore, it can be observed that the point cloud model exhibits a closer resemblance to the actual object model following the implementation of refraction compensation. This serves as empirical evidence supporting the efficacy of the compensation method. To a certain degree, it partially compensates for the limited study on the compensation algorithm employed in this particular dynamic refraction process. In addition, we have thoroughly analyzed the effects of physical noise, imaging distance, and partial obscuration of the measured target on the system in order to faithfully reproduce the underwater environment. The performance of the imaging device is constrained by factors such as the pixel resolution of the camera sensor and the mechanical inaccuracies of the driving mechanism. Within a specific range of measurements, the system exhibits high accuracy and imaging quality. However, outside of this range, the accuracy diminishes significantly. The FWLS scanning imaging transpose is less efficient than the pricey imaging system that uses the galvanometer scanning approach. Given the constraints of this publication, such as the limited imaging distance and overall working efficiency, future research could focus on enhancing both the imaging distance and working efficiency. Nevertheless, by employing the dynamic refraction compensation analysis method presented in this study, the error of the constructed simple imaging device is diminished by approximately 60%. Consequently, the investigation of compensating for underwater refraction errors holds significant scientific importance and provides reference value for future research on the dynamic refraction compensation method.

## Figures and Tables

**Table 1:** Analysis of standard ball error.

*D*/cm	*R*/mm	*Er*/mm	*Rw*/mm	*Er*/mm
30	18.87	−1.13	20.27	0.27
40	19.33	−0.67	20.68	0.68
50	18.76	−1.24	20.25	0.25
60	21.65	1.63	20.41	0.41
70	21.14	1.12	20.18	0.18
80	22.36	2.36	20.90	0.90

**Table 2:** Comparative analysis of parameters in industrial goods and relevant academic research.

	SXLS-100	ULS-100/200	FWLS	Xie [17]	Xue [14]
laser band (nm)	520 ± 10	520	520	530	530
Resolution (mm)	0.35/1	0.4/1	025/40		
Accuracy/Measurement distance (mm/cm)	0.5/60	0.4/1	0.49/40	0.7/60	0.6/120
Refresh rate of the scan line (HZ)	20~100	35	0.05~5	20~50	0.05~5
Measurement distance (cm)	30~100	13~100	30~150	50~100	50~120

## Data Availability

The data presented in this study are available from the corresponding author upon request.

## References

[1] Bianco G., Gallo A., Bruno F., Muzzupappa M. (2013). A Comparative Analysis between Active and Passive Techniques for Underwater 3D Reconstruction of Close-Range Objects. Sensors.

[2] Pang H.E., Biljecki F. (2022). 3D building reconstruction from single street view images using deep learning. Int. J. Appl. Earth Obs. Geoinf..

[3] Lau S.L., Lim J., Chong E.K., Wang X. (2023). Single-pixel image reconstruction based on block compressive sensing and convolutional neural network. Int. J. Hydromechatron..

[4] Massot-Campos M., Oliver-Codina G. (2015). Optical Sensors and Methods for Underwater 3D Reconstruction. Sensors.

[5] Malamas E.N., Petrakis E.G.M., Zervakis M., Petit L., Legat J.D. (2003). A survey on industrial vision systems, applications and tools. Image Vis. Comput..

[6] Nakatani T., Li S., Ura T., Bodenmann A., Sakamaki T. 3D visual modeling of hydrothermal chimneys using a rotary laser scanning system. Proceedings of the 2011 IEEE Symposium on Underwater Technology and Workshop on Scientific Use of Submarine Cables and Related Technologies.

[7] Shen Y., Zhao C., Liu Y., Wang S., Huang F. (2021). Underwater optical imaging: Key technologies and applications review. IEEE Access.

[8] Zhang X., Peng G., Chi T. Research on sea digital map used for ship navigation. Proceedings of the 2006 IEEE International Symposium on Geoscience and Remote Sensing.

[9] Qiu Y., Liu H., Liu J., Li D., Liu C., Liu W., Wang J., Jiao Y. (2023). A Digital Twin Lake Framework for Monitoring and Management of Harmful Algal Blooms. Toxins.

[10] Valkenburg R.J., McIvor A.M. (1998). Accurate 3D measurement using a structured light system. Image Vis. Comput..

[11] Geng J. (2011). Structured-light 3D surface imaging: A tutorial. Adv. Opt. Photonics.

[12] Liu J., Wang Y. (2021). 3D surface reconstruction of small height object based on thin structured light scanning. Micron.

[13] Chen C., Kak A. Modeling and calibration of a structured light scanner for 3-D robot vision. Proceedings of the 1987 IEEE International Conference on Robotics and Automation.

[14] Zhang Z. (2000). A flexible new technique for camera calibration. IEEE Trans. Pattern Anal. Mach. Intell..

[15] Dewar R. (1988). Self-Generated Targets for Spatial Calibration of Structured-Light Optical Sectioning Sensors with Respect to an External Coordinate System.

[16] Huynh D.Q., Owens R.A., Hartmann P. (1999). Calibrating a structured light stripe system: A novel approach. Int. J. Comput. Vis..

[17] Ma Y., Zhou Y., Wang C., Wu Y., Zou Y., Zhang S. (2022). Calibration of an underwater binocular vision system based on the refraction model. Appl. Opt..

[18] Xue Q., Sun Q., Wang F., Bai H., Yang B., Li Q. (2021). Underwater high-precision 3D reconstruction system based on rotating scanning. Sensors.

[19] Zhao J., Cheng Y., Cai G., Feng C., Xu B. (2022). Correction model of linear structured light sensor in underwater environment. Opt. Lasers Eng..

[20] Castillón M., Forest J., Ridao P. (2022). Underwater 3D scanner to counteract refraction: Calibration and experimental results. IEEE/ASME Trans. Mechatron..

[21] Xie Z., Li X., Xin S., Xu S. (2010). Underwater Line Structured-Light Self-Scan Three-Dimension Measuring Technology. Chin. J. Lasers.

[22] Wang H., Iwaguchi T., Kawasaki H. Robust calibration-marker and laser-line detection for underwater 3d shape reconstruction by deep neural network. Proceedings of the 2022 IEEE International Conference on Image Processing (ICIP).

[23] Ubiña N., Cai S.-Y., Cheng S.-C., Chang C.-C., Hsieh Y.-Z. Underwater 3D object reconstruction for fish length estimation using convolutional neural networks. Proceedings of the 2021 International Symposium on Intelligent Signal Processing and Communication Systems (ISPACS).

[24] Remondino F., Spera M.G., Nocerino E., Menna F., Nex F. (2014). State of the art in high density image matching. Photogramm. Rec..

[25] Nicolae C., Nocerino E., Menna F., Remondino F. (2014). Photogrammetry applied to problematic artefacts. Int. Arch. Photogramm. Remote Sens. Spat. Inf. Sci..

[26] Luhmann T., Fraser C., Maas H.-G. (2016). Sensor modelling and camera calibration for close-range photogrammetry. ISPRS J. Photogramm. Remote Sens..

[27] Maas H.-G. (1993). Robust automatic surface reconstruction with structured light. Int. Arch. Photogramm. Remote Sens..

[28] Bleier M., van der Lucht J., Nüchter A. (2019). SCOUT3D–An underwater laser scanning system for mobile mapping. Int. Arch. Photogramm. Remote Sens. Spat. Inf. Sci..

[29] Kwon Y.-H., Lindley S.L. Applicability of four localized-calibration methods in underwater motion analysis. Proceedings of the ISBS-Conference Proceedings Archive.

[30] Kwon Y.-H. (1999). Object plane deformation due to refraction in two-dimensional underwater motion analysis. J. Appl. Biomech..

[31] Fan H., Qi L., Ju Y., Dong J., Yu H. (2017). Refractive laser triangulation and photometric stereo in underwater environment. Opt. Eng..

[32] Ou Y., Fan J., Zhou C., Tian S., Cheng L., Tan M. (2023). Binocular Structured Light 3-D Reconstruction System for Low-Light Underwater Environments: Design, Modeling, and Laser-Based Calibration. IEEE Trans. Instrum. Meas..

[33] Zhang Z. (2004). Camera calibration with one-dimensional objects. IEEE Trans. Pattern Anal. Mach. Intell..

[34] Muralikrishnan B., Raja J. (2009). Least-squares best-fit line and plane. Computational Surface and Roundness Metrology.

[35] Yang Y., Zheng B., Zheng H.-Y., Wang Z.-T., Wu G.-S., Wang J.-C. 3D reconstruction for underwater laser line scanning. Proceedings of the 2013 MTS/IEEE OCEANS-Bergen.

[36] Zhu Z., Yang J., Wang X., Qi G., Wu C., Fan H., Qi L., Dong J. Rotation Axis Calibration of Laser Line Rotating-Scan System for 3D Reconstruction. Proceedings of the 2020 11th International Conference on Awareness Science and Technology (iCAST).

[37] Liu T., Wang N., Fu Q., Zhang Y., Wang M. Research on 3D reconstruction method based on laser rotation scanning. Proceedings of the 2019 IEEE International Conference on Mechatronics and Automation (ICMA).

[38] Wu Q., Li J., Su X., Hui B. (2008). An approach for calibrating rotor position of three dimensional measurement system for line structured light. Chin. J. Lasers.

[39] Yang H., Wang Z., Yu W., Zhang P. Center Extraction Algorithm of Linear Structured Light Stripe Based on Improved Gray Barycenter Method. Proceedings of the 2021 33rd Chinese Control and Decision Conference (CCDC).

[40] Li W., Peng G., Gao X., Ding C. (2020). Fast Extraction Algorithm for Line Laser Strip Centers. Chin. J. Lasers.

[41] Ma X., Zhang Z., Hao C., Meng F., Zhou W., Zhu L. An improved method of light stripe extraction. Proceedings of the Aopc 2019: Optical Sensing and Imaging Technology.

[42] Chadebecq F., Vasconcelos F., Lacher R., Maneas E., Desjardins A., Ourselin S., Vercauteren T., Stoyanov D. (2020). Refractive Two-View Reconstruction for Underwater 3D Vision. Int. J. Comput. Vis..

[43] Sun Q., Xue Q., Zhang D., Bai H. (2022). Research on the 3D laser reconstruction method of underwater targets. Infrared Laser Eng..

[44] Gu C.J., Cong Y., Sun G., Gao Y.J., Tang X., Zhang T., Fan B.J. (2022). MedUCC: Medium-Driven Underwater Camera Calibration for Refractive 3-D Reconstruction. IEEE Trans. Syst. Man Cybern.-Syst..

[45] Lyu N., Yu H., Han J., Zheng D. (2023). Structured light-based underwater 3-D reconstruction techniques: A comparative study. Opt. Lasers Eng..

[46] Li S., Gao X., Wang H., Xie Z. (2023). Monocular underwater measurement of structured light by scanning with vibrating mirrors. Opt. Lasers Eng..

[47] Castillón M., Palomer A., Forest J., Ridao P. (2021). Underwater 3D scanner model using a biaxial MEMS mirror. IEEE Access.

[48] Chantler M.J., Clark J., Umasuthan M. (1997). Calibration and operation of an underwater laser triangulation sensor: The varying baseline problem. Opt. Eng..

[49] http://www.hangzhoulanke.com/.

[50] https://voyis.com/insight-nano/.

